# Bacillus Typhosus in Sputum

**Published:** 1908-12-05

**Authors:** 


### PATHOLOGY.

### BACILLUS TYPHOSUS IN SPUTUM.

It may be thought, perhaps, that it is of little moment whether the lung complications that may arise in typhoid fever are due to the pneumococcus, or other superadded infecting organism, or whether they are caused by the bacillus typhosus itself. But if the vaccine treatment of lobar pneumonia becomes general, it will be of the greatest importance to know precisely what organism is at work in every inflamed lung. Moreover, although every possible precaution is presumably taken to disinfect anything that may have become contaminated by the bacillus typhosus in a case of typhoid fever, it is surprising what additional precautions may be found necessary when a fresh source from which the disease may be disseminated is discovered. If, for instance, a typhoid fever patient have a cough, and if it be known that the sputum never contains typhoid bacilli, it is quite clear that certain precautions against infection by particles of sputum might be less stringently insisted upon than would be the case if the sputum were known to contain the living typhoid bacilli in more than a few cases.

If only for these two reasons, therefore, the investigations that Dr. Stefanelli has been making in Florence are full of interest. He has made a study of the bacteriology of the bronchitis, broncho-pneumonia and lobar pneumonia that have occurred as complications in 15 out of a series of typhoid fever cases. In every patient Widal's reaction was positive, so that the diagnosis was not in doubt. As regards the varieties of lung complication, it took the form of broncho-pneumonia in 10 cases, lobar pneumonia in three cases, and severe bronchitis in two. The diplococcus pneumoniæ was present in the sputum in every one of the 15 cases, whilst the bacillus typhosus occurred also in six cases.

It is, of course, very difficult to say whether the diplococcus was causing the lung lesion in each instance or whether it was sometimes only accidental—normal sputum may contain diplococci. It is equally difficult to say whether the bacillus typhosus was merely undergoing excretion via the bronchi in the six cases, or whether it was really causing an inflammatory lesion in the lung. It is improbable that the bacillus typhosus would occur as an accident, in the way the diplococcus does; and it is well-established, from cases that have died and have been examined post-mortem, that not only may a pneumococcal pneumonia occur in the course of typhoid fever, but that there is also such a thing as a true typhoidal pneumonia due to the bacillus typhosus. If the patient recovers, it is naturally impossible to prove what organism caused the bronchitis or pneumonia, but Dr. Stefanelli's figures are none the less interesting in that they prove that typhoid fever patients suffering from lung complications may spread infection by reason of their sputa containing the bacillus typhosus.

